# Supplementary material for: RNA structure prediction using positive and negative evolutionary information
Source: PLoS Comput Biol. 2020 Oct 30;16(10):e1008387. doi: 10.1371/journal.pcbi.1008387 (PMC7657543; doi:10.1371/journal.pcbi.1008387)
Supplement: S1 File — Includes alignments, and source code. (GZ) [file pcbi.1008387.s008.tar.gz › supplemental_material/results/Figures/RF00020_U5.fold.R2R.sto.pdf]

RF00020_U5

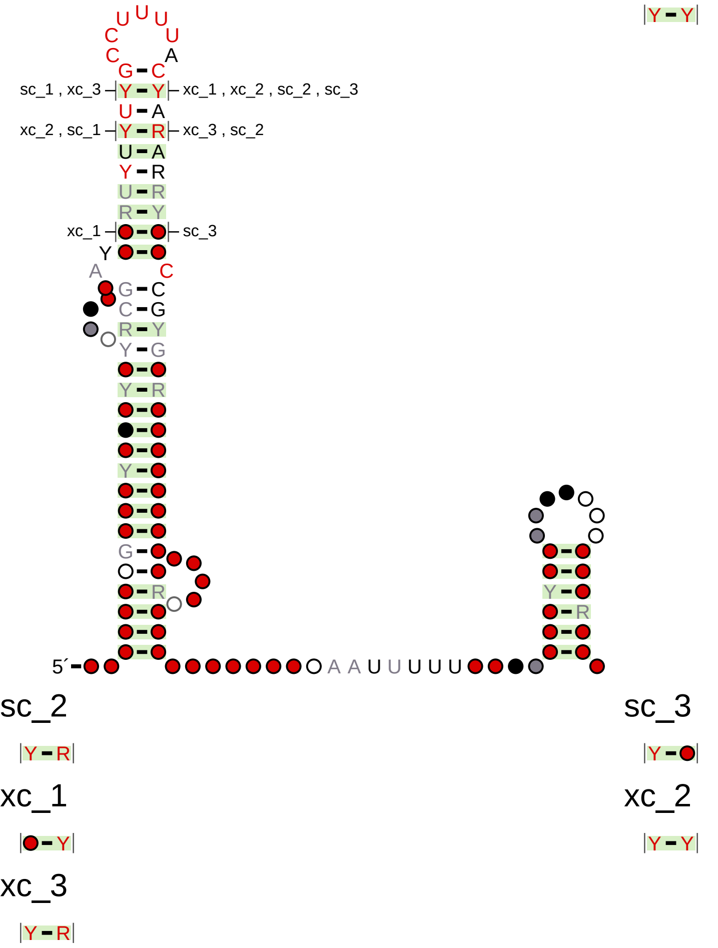

sc_1
sc_1 , xc_3
xc_1 , xc_2 , sc_2 , sc_3
xc_2 , sc_1
xc_3 , sc_2
xc_1
sc_3
5´
sc_2
sc_3
xc_1
xc_2
xc_3
